# Conceptualising Essential Oral Health Benefits Baskets: A Thematic Analysis of Public and Expert Perspectives

**DOI:** 10.1111/hex.70501

**Published:** 2025-11-20

**Authors:** Béatrice Durvy, Lukas Schöner, Tamara Reyes Ojopi, Katharina Achstetter, Reinhard Busse, Katherine Carr, Stefan Listl, Orsolya Németh, Amal Skandrani, Stéphanie Tubert‐Jeannin, Chris Vernazza, Juliane Winkelmann, Ruth Waitzberg

**Affiliations:** ^1^ Department of Health Care Management Technische Universität Berlin Berlin Germany; ^2^ European Observatory on Health Systems and Policies Brussels Belgium; ^3^ School of Dental Sciences Newcastle University Newcastle upon Tyne England UK; ^4^ Heidelberg Institute of Global Health Heidelberg University Hospital Heidelberg Germany; ^5^ Department of Dentistry – Quality and Safety of Oral Healthcare Radboud University Medical Center Nijmegen The Netherlands; ^6^ Department of Public Dental Health Semmelweis University Budapest Hungary; ^7^ Université Clermont Auvergne Clermont‐Ferrand France; ^8^ Myers‐JDC‐Brookdale Institute Jerusalem Israel

## Abstract

**Introduction:**

Despite its importance, oral health (OH) is often excluded from comprehensive public health coverage in Europe, resulting in financial hardship and exacerbating OH inequalities. Defining what ‘essential’ means in OH is a prerequisite for developing a public benefits basket to expand public coverage and financial protection.

**Method:**

This qualitative study explored the population's and experts' perspectives on ‘essential’ OH in Europe. Participants were purposively sampled from eight European countries. Data were collected through 37 focus group discussions (FGDs) gathering 228 participants. As data saturation was reached, data from 21 FGDs were analysed using thematic analysis.

**Results:**

‘Essential’ in OH was perceived as a state of good OH, encompassing both performing basic functions and the psychosocial dimension, which aligns with the WHO definition of good OH. Participants highlighted multiple considerations to define an ‘essential’ OH benefits basket, including services' contributions to improving health, economic criteria, societal values, people‐centred care and feasibility. Considering OH as part of a broader health system was seen as crucial.

**Conclusion:**

Findings emphasise the fluid and multidimensional nature of the ‘essential’ concept in OH and highlight the myriad considerations for priority‐setting in public coverage. This underscores the importance of first defining what is ‘essential’ in each country's context. This study identifies three key lessons relevant for policy‐making. First, people's expectations vary between and within countries but remain realistic, as considerations are similar to those used in other health fields. Second, OH should be better integrated into the general health system. Finally, involving patients and potential patients in the decision‐making process is key to (re‐)defining an OH benefits basket that responds to the health system's goals.

**Public Contribution:**

OH patients and potential patients were at the centre of this study, informing how to conceptualise ‘essential’ in OH in Europe. This is a concrete example of how public participation mechanisms can support decision‐making over the definition of an (OH) benefits basket.

## 1 Introduction

Achieving the highest attainable standard of oral health (OH) is a fundamental human right [1], as OH is key to overall health and well‐being and considered an important marker of social progress [2]. The World Health Organization (WHO) defines good OH as ‘the state of the mouth, teeth and orofacial structures that enables individuals to perform essential functions such as eating, breathing and speaking, and encompasses psychosocial dimensions such as self‐confidence, well‐being and the ability to socialize and work without pain, discomfort and embarrassment’ [1]. Yet, the burden of oral diseases is a substantial public health challenge, which affects 3.7 billion people worldwide [3, 4]. Social determinants of health, such as socioeconomic or employment status, influence the prevalence of oral diseases and access to OH care. Vulnerable populations often bear a disproportionate burden of oral diseases and treatment costs [5, 6], with a reported 13 percentage points difference in unmet dental care needs in the European Union (EU) between the lowest income quintile (15%) and the highest quintile (2%) in 2023 [7].

Limited OH coverage is a key driver of financial hardship in Europe [8]. Although EU countries are aiming for universal health coverage (UHC), OH care is often only partially covered by public health systems, with wide variations across countries and population groups [9]. Out‐of‐pocket (OOP) payments accounted for 57% of total OH spending on average in the EU in 2022, representing the largest source of OH funding in most European countries. This share varies widely between EU countries, ranging from 11% in Ireland to 100% in Greece. Another 9% of OH spending is financed privately via voluntary health insurance [10]. In 2019, expenditure on OH care represented on average 14.2% of households' OOP health expenditure in the EU [9] and OOP payments on dental care are among the main drivers of catastrophic spending in all income groups [8].

Efforts to improve OH coverage and OH care integration within public health systems generally gained momentum in recent years [11]. The WHO is advocating for countries' greater interest in addressing OH needs, the definition and consolidation of an essential OH benefits basket, and the better integration of the OH system in the primary healthcare system [1, 11]. At the national level, some countries are expanding their public coverage of OH. For example, Portugal actively integrated OH care within primary healthcare delivery [12] and France expanded OH coverage with its ‘100% santé’ reform [13].

Nonetheless, when (re‐)shaping public coverage, careful consideration must be given to financial and human resources, with priority placed on services deemed ‘essential’. Conceptualising what constitutes ‘essential’ OH care is therefore critical for shaping a (public) OH benefits basket, but there is a literature gap regarding how ‘essential’ is conceptualised in the OH context. Furthermore, UHC goal is to enable everyone's access to quality health care where and when needed and without financial hardship [14], which places individuals at the heart of health systems' objectives. Hence, there is a compelling case for involving patients and potential patients in the development of public coverage policies for OH. This paper aims to inform European decision‐makers about the population's and experts' shared understanding of what constitutes ‘essential’ OH care. This study also illustrates how participatory decision‐making, engaging both the general population and providers, can support more inclusive health policy‐making. Through qualitative research in selected European countries, this study explores the perspectives of the general population and experts on how ‘essential’ OH is defined, how an ‘essential’ OH benefits basket is characterised and what ‘essential’ OH requires beyond OH coverage.

## 2 Methods

Drawing from a qualitative approach, this study explored the views of the general population and experts on how to conceptualise ‘essential’ in OH through focus group discussions (FGDs). This study is part of the PRUDENT project, an EU project funded by the European Commission under the Horizon Europe programme (Grant Agreement Number: 101094366). The project uses a transdisciplinary research approach to develop and implement an innovative and context‐adaptative framework for optimal financing of OH care enhancing equitable access to essential OH care for all [15].

The overall purpose of this task of the PRUDENT project was to identify a common understanding of European stakeholders' perspectives on what constitutes an essential OH benefits basket. Hence, the primary objective of this study was to better understand the general population's (understood as both patients and potential patients) and OH experts' opinions on what is ‘essential’ OH. Secondary objectives were to capture participants' perspectives on considerations for defining ‘essential’ OH services for a public benefits basket and what is essential to good OH beyond public coverage.

The study, data collection tool and methods received ethics approval from Newcastle University (Ref: 33388/2023; see Annex S1). All participants read and signed an informed consent form agreeing to participate prior to the FGD (see Annex S2).

### 2.1 Setting, Sampling and Recruitment of Participants

To expand heterogeneity in the perspectives on what is deemed essential in OH, a two‐step maximum variation sampling strategy [16] was applied.

In a first step, eight European countries (i.e., Denmark, Estonia, France, Germany, Hungary, the Netherlands, Portugal and the United Kingdom (England)) were sampled to include a variety of health systems characteristics and resource levels (public spending on health as a share of GDP and per capita; rates of health workers per capita).

In a second step, participants were purposefully sampled into two population sub‐groups, that is, the general population and (oral) health experts. The first group was constituted of individuals from the general population from a subset of countries (i.e., France, Germany, Hungary and the United Kingdom (England)), including both users (i.e., patients) and those who may forgo OH care (i.e., potential patients). Recruitment strategies included snowball recruitment via students, associated hospitals and panels.

The second group was constituted of experts recruited within the PRUDENT consortium from another subset of countries (i.e., Denmark, Estonia, France, Hungary, the Netherlands, Portugal and the United Kingdom (England)). This method is appropriate for identifying and selecting individuals who are especially knowledgeable about or experienced in the subject matter [17].

Participants were purposefully sampled based on the following criteria:

▪ Inclusion criteria: aged 18+, who are eligible for OH care publicly covered by the health system in their country of residence and who give informed consent.
▪ Exclusion criteria: younger than 18, those not publicly covered, those who are not able to communicate in the language of the FGD and those unwilling or unable to give informed consent.

**Figure 1:**
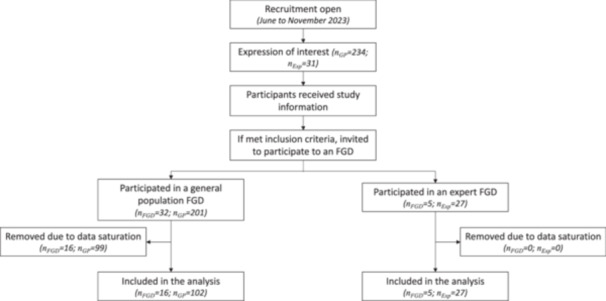
Participants flow chart. FGD, focus group discussion; *n*_GP_, number of individuals from the general population group; *n*_Exp_, number of individuals from the expert group; *n*_FGD_, number of focus group discussions.

Overall, 228 persons participated in a total of 37 FGDs. FGDs were first run among experts, followed by the general population in Germany, and finally, FGDs among the general population in England, France and Hungary, which were conducted in parallel. Data collection was conducted for multiple aims, hence data saturation was assessed at two levels. First, a preliminary analysis of the FGD transcripts of the general population groups in Germany and the expert groups was run in parallel to the data collection in England, France and Hungary. Data collection was stopped once data saturation in the analysis was reached. Second, data from both group categories and in every country were analysed, and the data analysis was stopped once no new information was identified. Consequently, all transcripts from the expert groups and the general population groups from England, France and Germany were included, but 16 out of 23 FGDs from Hungary were excluded due to data saturation. Hence, a total of 21 FGDs were included in the analysis of this study, comprising 16 among the general population and 5 among experts (Figure 1).

Included FGDs account for the participation of 129 individuals, who were split into 16 general population FGDs that comprised a total of 102 participants, and 5 experts FGDs that comprised 27 participants. The average number of participants per FGD was 6.4 in the general population groups and 5.4 participants in the expert groups. In Germany and Hungary, six or more general population FGDs were conducted. In contrast, in France and the United Kingdom only one and two, respectively, general population FDGs were conducted (Table 1).

**Table 1:** Participants and FGD included in the analysis.

Country of residence		DE	DK	EE	FR	HU	NL	PT	UK^a^	Sub‐total	Total
Number of participants included	General population	39	—	—	6	43	—	—	14	102	129
	Experts	—	2	2	5	3	7	3	5	27	
Number of FGDs included	General population	6	—	—	1	7	—	—	2	16	21
	Experts	NA	NA	NA	NA	NA	NA	NA	NA	5	

*Note:* Only the FGDs and participants characteristics of the FGDs included in the analysis are depicted in the table.

Abbreviations: FGDs, focus group discussions; DE, Germany; DK, Denmark; EE, Estonia; FR, France; HU, Hungary; NL, Netherlands; PT, Portugal; UK, United Kingdom; NA, not applicable.

a Participants from the general population included in the study are residing in England only.

The general population and experts' groups were both composed of around two‐thirds of women (Table 2). Participants from all age groups, except under 18 years old, were included, even though the general population group was mostly composed of participants aged between 18 and 29 years old and the expert group was mostly composed of participants aged 30 years old and above. Experts included in the study were OH professionals (e.g., dentists, dental hygienists) and health systems experts (e.g., health economists, legal specialists, public health researchers) (Table 3). To maintain anonymity, experts' characteristics and quotes are not depicted by country of residence.

**Table 2:** Participants' gender and age characteristics.

	Experts	General population	Participants' country of residence and location of FGDs				Total^a^
			England	France	Hungary	Germany	
Gender, *n* (%)							
Male	9 (33)	21 (33)	7 (50)	1 (17)	13 (30)	na	30 (33)
Female	18 (67)	42 (67)	7 (50)	5 (83)	30 (70)	na	60 (67)
Age group, *n* b							
18–29	2	39	3	4	32	na	41^c^
30–49	14	10	3	2	5	na	24^c^
50–69	10	11	5	0	6	na	21^c^
70+	0	3	3	0	0	na	3^c^

*Note:* Only the participants characteristics of the FGDs included in the analysis are depicted in the table.

Abbreviations: FGDs, focus group discussions; *n*, number of participants; %, percentage of participants; na, not available.

a Include available participants' characteristics for both the general population and experts.

b Data for one participant from the expert group and data for the general population groups conducted in Germany were not collected.

c Total of the available data.

**Table 3:** Experts field of expertise.

Background	Number of experts holding this background
Oral hygienist	1
Dentist	11
Specialised dentist	2
Public health researcher	5
Health economist	9
Health system specialist	1
Legal specialist	4

*Note:* Some experts hold multiple expertise.

### 2.2 Data Collection

Data collection took place between June and November 2023. Data were collected through FGDs using an open‐ended topic guide (see Annex S3) developed based on two frameworks: the scope and breadth dimension of the ‘universal health coverage cube’ from Winkelmann et al. [18] and the classification of OH services from Winkelmann et al. [9]. The topic guide included four questions. The first two related to the concepts of ‘what is essential for OH’ and ‘what is an essential OH service’. For the last two, participants were shown a table depicting categories of OH care and were asked to select the services and/or population groups considered essential and/or the most important to cover for/be covered. The topic guide was developed under an iterative process, which included slight amendments following the first session of FGDs, and was adapted according to the target audience. FGDs were conducted with an average duration of 1 h. FGDs with the general population were conducted in English, French and Hungarian (in England, France and Hungary, respectively) by national investigators from the PRUDENT consortium. FGDs in Germany were conducted in English, but participants were allowed to contribute in German if specific concepts or terminology were difficult to express in English. FGDs with the experts were conducted in English. FGDs with the general population were conducted in‐person except in England and France, where they were conducted online (via Zoom or Microsoft Teams), and FGDs with the experts were also conducted online. All FGDs were recorded, transcribed verbatim and manually validated. Data were transcribed using Notta software, Alrite software, Whisper software or Zoom. Transcripts in French and Hungarian were translated into English using DeepL, followed by a quality check by native speakers. Participants from the general population in England were offered a voucher of EUR 25 each for their participation. All other participants did not receive any compensation.

### 2.3 Data Analysis

Data were analysed through a thematic analysis conducted with ATLAS.ti 22 software. Thematic analysis of the data involved familiarisation with the transcripts, coding and building themes [19]. Intercoder validity was applied throughout the whole process: all transcripts were read, analysed and coded independently by four researchers, who then cross‐validated and reconciled the coding. The analysis approach was both deductive, as codes and categories were initially built from the research questions and based on the WHO health systems' building blocks analytical framework [20], and inductive, as they were further refined, deleted and created based on participants' narratives and discourse. Overall, a reflexive approach, allowing for flexible, organic and evolving coding, was applied to analyse the FGD transcripts [21]. All codes were discussed, compared and further developed repeatedly between the involved four researchers.

## 3 Results

**Figure 2:**
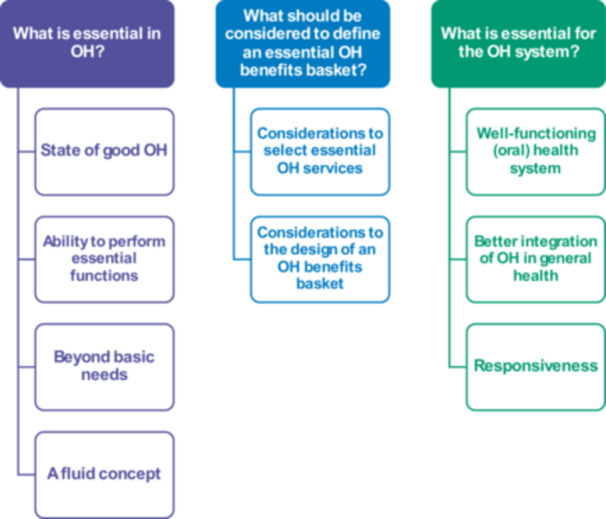
Themes and categories that conceptualise ‘essential’ in relation to OH. OH, oral health. *Source* : Authors.

A total of 139 codes were identified and grouped into three themes: ‘What is essential in OH?’, ‘What should be considered to define an essential OH benefits basket?’ and ‘What is essential for the OH system?’ (Figure 2). For each of these themes, categories were formed to capture the results of the FGDs.

### 3.1 Theme 1: What Is Essential in OH?

**Figure 3:**
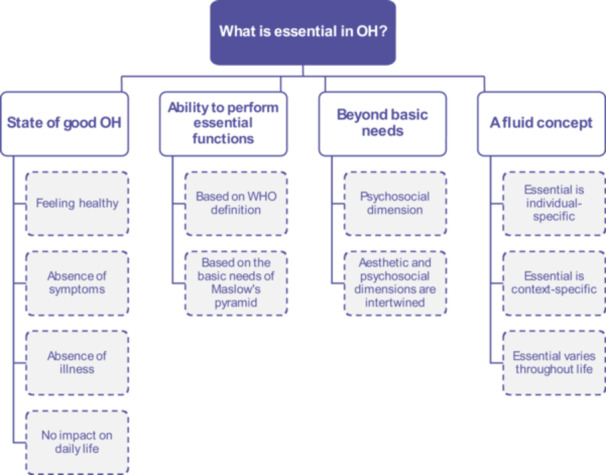
Categories and sub‐categories regarding ‘What is essential in OH?’. OH, oral health. *Source*: Authors.

When conceptualising what ‘essential’ means in the OH context, participants' answers and ideas were clustered into four main categories, with two to four sub‐categories (Figure 3).

Participants described ‘essential’ in OH as a state of good OH, meaning feeling healthy: ‘I want to stay as healthy as possible for as long as possible’ [General population, Hungary], or the absence of symptom and/or illness or impact on daily life: ‘[It is essential that] if I want to do something [I am] able to do [it] without any limitations’ [General population, UK].

Individuals' need to maintain the ability to perform essential functions was highlighted, sometimes referring directly to the WHO definition of good OH. Some participants described these ‘essential’ functions in direct relation to the Maslow's pyramid of needs hierarchy (see Section Conclusion, 4), while others referred to ‘basic or survival needs’: ‘I was wondering whether essential means that I need it? That I really have to have this to maybe survive’ [General population, Germany].

Yet, all groups also mentioned that good OH goes beyond basic needs, particularly the impact of oral aesthetics on how individuals perceive themselves, ‘self‐esteem considerations are very important when it comes to the mouth’ [General population, France]. Participants also argued that poor oral aesthetics can negatively affect how an individual is perceived by others, potentially leading to stigmatisation, discrimination and difficulties in participating in society, ‘there is an inherent discrimination element towards people with bad teeth’ [Expert]. Furthermore, participants highlighted that psychosocial wellbeing is intertwined with the aesthetic of the mouth in a two‐way relation, which could lead to either a virtuous or vicious circle, as per this example of the potential negative consequences of a poor mouth aesthetic: ‘Mental health gets bad because someone isn't feeling comfortable with their oral aesthetics. In return, the person might not take care of their oral health. So, it further declines.’ [General population, Germany]. However, despite a general consensus that ‘essential’ OH goes beyond basic functions, debates arose in many groups about how to balance between accounting for individuals' psychosocial needs while not wasting public resources on non‐essential cosmetic care.

Participants further recognised the fluidity of the concept of ‘essential’ in OH. For example, essential was seen as individual‐specific, meaning that individuals consider essential differently according to their priorities, needs or characteristics: ‘It can't be [one] rule for everybody. It should be tailor‐made.’ [General population, Germany], further varying across individuals' life course. Moreover, essential OH is context‐specific: ‘Essential differs between different parts of the society and in different countries too’ [Expert], which may also vary across time, ‘the answer we are giving today would have probably been different three or four years ago’ [Expert].

### 3.2 Theme 2: What Should be Considered to Define an Essential OH Benefits Basket?

**Figure 4:**
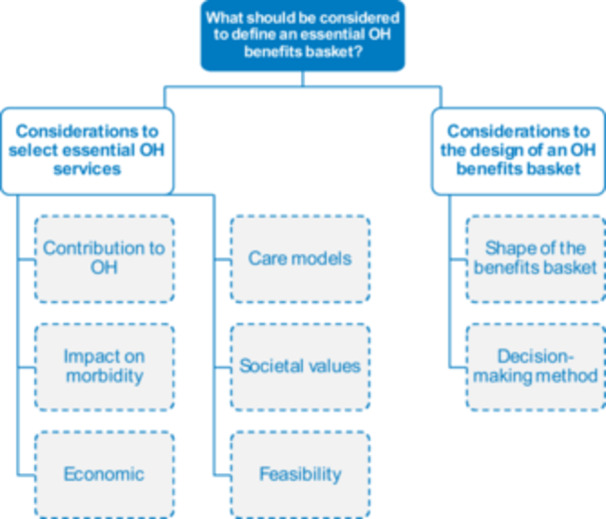
Categories and sub‐categories regarding ‘What should be considered to define an essential OH benefits basket?’. OH, oral health. *Source*: Authors.

Conceptualising what is an ‘essential’ OH service is the first step to define an essential OH benefits basket. Two categories of considerations to define an OH benefits basket were identified, namely considerations to select the essential services and considerations to design an OH benefits basket (Figure 4).

When asked what ‘essential’ means in OH, participants often responded with services that they considered essential. To better understand what is an ‘essential OH service’ and why these services are ‘essential’, we disentangled participants' considerations when defining a service as essential.

The first consideration was its contribution to an individual's OH by either maintaining the overall OH or the essential functions of the mouth, ‘the primary aim [of OH care] should be to preserve health’ [General population, Hungary]. Others set a lower threshold defined by the safety of OH services, ‘if there is clear evidence that [an OH service] is doing more harm than good, then [it] should be excluded’ [Expert].

The impact of a service on reducing morbidity at the populational level was commonly mentioned as a consideration to define it ‘essential’. Hence, services that tackled high‐incidence diseases, such as tooth decay or periodontal diseases, were deemed essential, ‘at the level of society as a whole, when examining [OH] financing, it is necessary to look at the incidence of these diseases’ [General population, Hungary], and participants referred to the broader idea that essential services are services that benefit most people.

Economic considerations were mentioned as well. Specifically, cost‐effectiveness, such as ‘detecting early some diseases or problems can save health expenditure later on’ [General population, Germany]; marginal return or ‘maximize health gain: essential should be defined as to the [patients] in need, but with the largest potential gain from receiving services’ [Expert]; and willingness to pay for OH services. The value of shifting from curative care to preventive care was also particularly emphasised as a potential cost‐saving strategy for OH care and all groups generally agreed on enhancing the focus on preventive services. Some participants also highlighted that accounting for the quantity of care (e.g., number of preventive check‐ups per year per individual) and the quality or types of material used (e.g., types of filling used) is particularly key when defining an essential OH benefits basket as it impacts individuals' OH outcomes, as well as both private and public costs.

Considerations around levels and models of care were at the heart of many discussions. The concept of population‐based care was mentioned and mostly agreed on, ‘we want to, of course, ensure that the distribution of healthcare is based on population need’ [Expert]. At the same time, care at the individual level was recognised as key, including the concept of people‐centred care, ‘the most important is that [OH care] is individualised and tailored to each individual's needs’ [General population, Hungary]. Participants also highlighted the important role of individual responsibilities and behaviours on their own OH, as eluded by one of the experts: ‘If you've been given all the advice in the world on how to brush your teeth and still don't do it, then should you have priority?’ [Expert]. While some participants emphasised individuals' responsibility over their own OH, others strongly argued against it, stressing the impact of social determinants on individuals' health behaviours and the perception of their responsibility for their health.

Societal values were important considerations when defining ‘essential’ OH services, including the need to account for the impact of social health determinants on OH. Principles of equity and solidarity that are at the heart of many EU health systems were also mentioned as important considerations, and participants suggested that ‘obviously financial situation can be a good starting point [for prioritisation] by covering those who are more disadvantaged’ [General population, Hungary].

Finally, the feasibility of ensuring public coverage for certain services or of applying certain coverage mechanisms was also identified as important when defining an ‘essential’ OH benefits basket. For example, participants emphasised that decision‐makers should consider the level of availability of resources, may they be human, material, or financial, ‘one big problem is that resources are obviously becoming scarcer and the demand for care is increasing, so sustainability is the issue’ [General population, UK]. Other feasibility considerations included the difficulty of identifying who is financially and/or socially vulnerable and who needs better access, the notion of accessibility, or the challenge of assessing the impact of OH conditions on individuals' psychosocial health.

While reflecting about how to select what should be included in an essential OH benefits basket, experts further referred to the decision‐making process. Beyond questioning what makes an OH service essential, participants emphasised that OH benefits baskets could take various shapes, such as a positive or negative list of services, eligibility criteria or beneficiaries, or could even be based on care pathways, ‘can we think of the question [of public coverage] without considering lists of services?’ [Expert]. Experts also reflected on how granular the OH benefits basket should be, ‘even under the same category, there might be some services that are more or less essential’ [Expert]. Finally, participants referred to the decision‐making process such as the need of evidence‐based decision‐making, and involving all concerned stakeholders when defining an adequate OH benefits basket, ‘listen to citizens and understand from them what they find relevant’ [Expert].

### 3.3 Theme 3: What Is Essential for the OH System?

**Figure 5:**
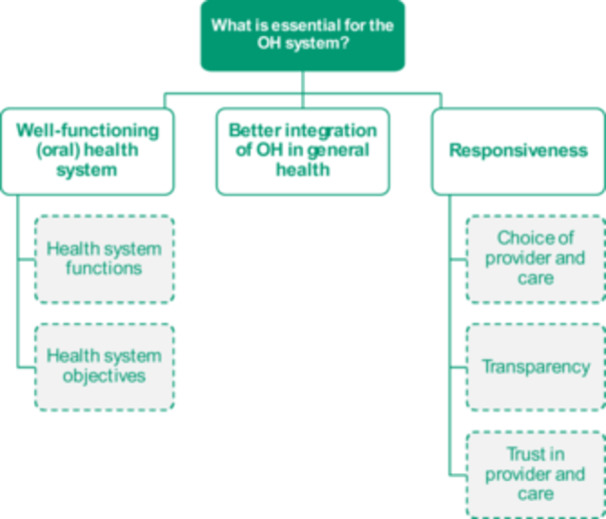
Categories and sub‐categories regarding ‘What is essential for the OH system?’. OH, oral health. *Source* : Authors.

Conceptualising ‘essential’ in OH also includes the health system in which OH care is built. Three categories were identified, some of which were further divided into two to three sub‐categories (Figure 5).

Participants' input highlighted the importance of a well‐functioning OH system that offers timely access to high‐quality care. Participants answers (directly or indirectly) referred to several building blocks of the WHO health system framework [20] functions as essential for the OH system, as expressed: ‘I think it is about making sure that you only have the treatment that you need, when you need it, and that it is delivered by the right person’ [General population, UK]; as well as the health system objectives, ‘[OH] has to be affordable’ [General population, UK].

Furthermore, throughout the FGDs, participants referred to the need for OH to be better integrated within the general health system, emphasising the importance of a unified essential benefits basket, ‘We need to move to a point where we include OH in our considerations of overall healthcare and how much are we willing to pay for our healthcare system. Our willingness to pay shouldn't be for general healthcare separately and then for oral healthcare separately.’ [Expert].

Finally, several elements of health systems' and providers' ‘responsiveness’, as defined by the WHO [20], were deemed particularly important in the context of OH and key to promote patients' adherence to effective care. Some participants referred to aspects of responsiveness related to the choice of provider and inclusion in care decision‐making, ‘it is important that care is aligned with patients wishes’ [General population, France]. Others alluded to transparency in care costs, treatment options and providers qualifications. Trust was seen as essential in OH as a mean to reduce fear, which is often a barrier to acceptability of care, and can have lifelong impacts: ‘Early negative experiences in childhood lead to a decrease in the frequency of visits to the dentist in later life. So, if we can gain the patient's trust, they will probably pay [more] attention to visiting the dentist [regularly].’ [General population, Hungary].

## 4 Discussion

### 4.1 Interpretation

This study aimed to explore how Europeans conceptualise ‘essential’ in the context of OH. Based on data collected through FGDs, three main themes were identified: conceptualising what is ‘essential’ in OH, what to consider when shaping an ‘essential’ OH benefits basket and what is ‘essential’ for the OH system. Key elements to define ‘essential’ in OH are in line with the WHO definition of good OH [1], which describes a state of well‐being and absence of illness or symptoms that encompass basic needs related to the essential functions of the mouth, as well as more complex needs linked to individuals' psychosocial well‐being. Findings unveil the fluidity of the concept of ‘essential’ in OH, as its definition may vary across people, countries and time. Key considerations to the selection of essential OH services include services' safety and contribution to maintaining or improving OH, prioritising services that benefit to the most, economic considerations, societal values, people‐centred care and feasibility. An essential OH benefits basket can take multiple shapes and could be rethought to be designed around care pathways rather than OH service lists. Participants also reaffirmed the importance for better integration of the OH system into the general health system. Findings are aligned with WHO principles of health benefits baskets [22]: decision‐making should be evidence‐based, transparent, inclusive and realistic; the benefits basket and the criteria to define it should reflect the country's core values and principles; and the effectiveness of a public benefits basket relies on broader considerations at the system level. Hence, three key lessons relevant for policy‐making can be derived from the findings.

### 4.2 People's Expectations Vary but Are Realistic

While most European countries commit to only a minimum level of basic OH care, such as routine oral examinations, X‐rays, fillings or tooth extractions [9, 11, 23, 24], the process through which OH benefits basket in Europe are built is not always known, often resulting from historical developments, incremental extensions or negotiations, rather than evidence‐based decision‐making [25, 26]. This results in wide variations across countries of the (partial) coverage for OH care [9].

Unlike OH, benefits baskets for other healthcare areas are mostly built using evidence‐based technology assessments that highlight economic criteria such as cost‐effectiveness [27]. Yet, individuals' decision to use health services, including OH care, is multifactorial, with choices not necessarily based on costs, professionals' advice or treatment outcomes [25]. Previous work on other healthcare areas also highlights the importance of considering broader sets of factors when deciding on ‘essential’ care. For example, an ‘essential medicines list’ aims not only for economic criteria related to better affordability, improved cost‐effectiveness and price reductions, but also considers broader criteria such as health equity, burden of disease, or timeliness of treatment [28]. These sets of criteria could be implemented for OH. Moreover, Vernazza and colleagues recently studied the feasibility of integrating willingness‐to‐pay considerations, which account for the population's values, as well as broader criteria, which goes beyond cost and outcome, into OH resource allocation processes; therefore showcasing innovative wider approaches to decision‐making in OH [29].

In a context of thin evidence on effectiveness of many OH services [30] and as the definition of ‘essential’ is fluid and varies across people, countries and time, this study highlights Europeans' considerations for prioritisation and defining an ‘essential’ OH benefits basket. While economic and feasibility considerations were raised, other considerations were also regarded such as clinical benefits, equity and solidarity and recognising the impact of social determinants of (oral) health.

Many participants suggested considering innovative care approaches when defining an essential benefits basket, hence emphasising the need for individualised care that responds to populations' needs. A people‐centred approach is a multidisciplinary concept, which consist in placing patients at the centre of care and treating them as equal partners in the care process [31], and population‐based care is another innovative approach that focuses on the health outcomes of a population group or community [32]. These concepts are gaining interest in primary healthcare reforms and OH could be included when rethinking approaches to care, especially if shifting the focus from curative to preventive care.

Hence, there is a need for a more holistic approach when defining OH benefits basket in Europe, which further emphasises the importance for decision‐makers to have a good understanding of the population's views on their OH needs. Findings suggest that although people's expectations vary between and within countries, considerations for decision‐making are similar to those that European countries are already using in other health fields. While this might call for sometimes drastic change, it also suggests that these are realistic expectations.

### 4.3 OH Could Be Better Integrated Into the General Health System, Particularly in Primary Care

While the OH system lacks integration into the general health system, OH and general health are strongly intertwined [1]. There cannot be good health without good OH nor there can be UHC without OH coverage [1, 33]. Findings show that people indeed expect the OH system to be better integrated within the general health system. Participants emphasised that a well‐functioning OH system is no different than the general health system. OH systems share the same objectives of access, coverage, quality and safety of OH care, as well as goals of improved health level and equity, responsiveness, including transparency, and social and financial protection [20]. In particular, in the domain of responsiveness, trust is key both at the population and individual levels, especially users' trust in OH care and in their OH professionals [25]. Moreover, the 2019 Lancet commission on OH advocated for a better integration of OH care into primary healthcare [34] and the WHO set a target for 80% of the countries in the WHO European region to have integrated OH into primary healthcare by 2030 [1]. Our findings highlight that both the general population and experts support such integration, and even consider it ‘essential’ in the context of OH.

### 4.4 Embedding People's Expectations in Decision‐Making of OH Coverage Policies

This study shows that there was a general consensus among participants that good OH is essential, encompasses both essential functions and the psychosocial dimension, and essential care should be accessible to everyone. Nonetheless, participants also recognised the fluidity of the definition of ‘essential’ OH and multiple debates arise. Conversations on the trade‐off between covering for cosmetic care that respond to psychosocial needs while deprioritising ‘comfort’ care, as well as debates on individual responsibility versus the consequences of social health determinants, illustrate the complexity of balancing individual essential needs and the overall population's best interest, especially in a context of limited resources. Hence, the absence of a shared normative understanding about the specific types of OH care that are essential to cover combined with the importance for this benefits basket to reflect countries' core values and principles, constitute a strong argument for better involving beneficiaries in the decision‐making process of (re‐)designing an OH benefits basket.

Social participation is an umbrella concept, which recently gained global attention, and emphasises the inclusive and empowering participation of people, communities and civil society in strengthening UHC and policy making [35, 36]. In particular, the unanimous adoption of the World Health Assembly resolution on social participation urges all WHO Member States to take concrete actions towards embedding and sustaining social participation into their health systems' modus operandi [37]. Applicable to all spheres of decision‐making, from the design of overarching health strategies to the implementation of targeted interventions, social participation should be at the heart of the discussions on conceptualising ‘essential’ benefits basket and defining OH coverage in Europe. Social participation is a win–win practice that not only ensures that people's voices, real‐world needs and concerns are heard and accounted for, but also represents a unique opportunity for policy‐makers to foster collaborative policy implementation and thereby trust and transparency [38, 39, 40]. By contributing to population's better understanding of the prioritisation choices made, social participation can enhance trust and transparency [41], two key goals of the OH system, as well as increase equity and efficiency by enabling strong collaboration and engagement of all stakeholders [42].

This study supports that social participation mechanisms are key to (re‐)shape OH coverage across Europe, as it can be a crucial asset to both policymakers and the population. Findings highlight that prior to defining a benefits basket, it is important to learn people's expectations for their OH (care) and understand the definition of ‘essential’ OH in the country context. It also further emphasises the need to involve all stakeholders in the decision‐making and argues that rather than ad hoc integration, people's expectations and needs could be the starting point of the prioritisation task. Hence, this study provides key insights for including collaborative mechanisms in the design of an OH benefits basket and illustrates how to operationalise social participation to inform decision‐making on OH coverage.

## 5 Limitations and Strengths

Despite its unique findings and clear derived recommendations, the study has some limitations. The sample includes a limited number of countries, and not all European countries. Countries included were sampled to add variation in perspectives within Europe, yet a more diverse sample could have rendered a richer set of perspectives. Age was not collected for a small number of participants. In groups with age data, young people are overrepresented, as well as women. Nonetheless, representativeness of the country population was not sought, but rather a broad variety of opinions across Europe. Here, a diverse set of country of residence and all age categories (i.e., 18–29 years old; 30–49; 50–69; 70 and older) are purposefully selected, enabling to create a holistic picture of opinions from a broad range of population groups. Furthermore, data saturation was reached, indicating that the findings are likely to be exhaustive for the research question.

In line with the overall purpose of this study to identify a common understanding of European stakeholder perspectives on what constitutes an essential OH benefits basket, no country‐specific analysis was conducted to consider different underlying health system‐related reasoning. Neither sub‐group analysis comparing the answers from the general population and experts' groups was conducted. While this would be an interesting follow‐up study, it is out of the scope of the current work, which was to obtain general implications for policymakers at the European level that can be applied by national decision‐makers within their country context. Future work, including in forthcoming workstreams of the PRUDENT project, is warranted to complement the findings of the present study with grounded theory, as well as quantitative studies, to understand the importance of each theme and category, or the extent to which the different perspectives represent the European population.

## 6 Conclusions and Policy Recommendations

This qualitative study explored how Europeans conceptualise ‘essential’ in the context of OH. The described fluidity of the concept of ‘essential’ coupled with the myriad of considerations for priority setting, illustrate the importance of first defining what is ‘essential’ in OH in the country context. Findings align with the WHO's principles guiding the consolidation of health benefits basket and three lessons relevant for policy‐making can be derived. First, people's expectations vary between and within countries but remain realistic. Considerations for decision‐making about an OH benefits basket are similar to those that European countries are already using in other health fields, such as contribution to maintaining or improving OH, prioritising services that are a benefit to most people, economic considerations, societal values, people‐centred care and feasibility. Second, OH should no longer be thought of as outside of the general health system. There is no health without OH, and there is no UHC without OH coverage. Finally, social participation is key to the process of (re‐)defining an OH benefits basket. Enhancing the population's participation in decision‐making processes to define public coverage can be a crucial asset to both policymakers and the population. Explorative research initiatives such as this study can be concrete examples of how to integrate people's views into the conceptualisation of ‘essential’ OH and the definition of an OH benefits basket.

## Author Contributions

**Béatrice Durvy:** data curation, formal analysis, investigation, methodology, project administration, supervision, validation, visualisation, writing – original draft, writing – review and editing. **Lukas Schöner:** data curation, formal analysis, investigation, methodology, project administration, validation, visualisation, writing – original draft, writing – review and editing. **Tamara Reyes Ojopi:** formal analysis, project administration, validation, visualisation, writing – original draft, writing – review and editing. **Katharina Achstetter:** validation, writing – original draft, writing – review and editing. **Reinhard Busse:** conceptualisation, funding acquisition, methodology, project administration, supervision, validation, visualisation, writing – original draft, writing – review and editing. **Katherine Carr:** investigation, writing – review and editing. **Stefan Listl:** conceptualisation, funding acquisition, writing – review and editing. **Orsolya Németh:** investigation, writing – review and editing. **Amal Skandrani:** investigation, writing – review and editing. **Stéphanie Tubert‐Jeannin:** investigation, writing – review and editing. **Chris Vernazza:** conceptualisation, funding acquisition, investigation, writing – review and editing. **Juliane Winkelmann:** conceptualisation, funding acquisition, writing – review and editing. **Ruth Waitzberg:** data curation, formal analysis, investigation, methodology, project administration, supervision, validation, visualisation, writing – original draft, writing – review and editing.

## Ethics Statement

The study, data collection tool and methods received ethics approval from Newcastle University (Ref: 33388/2023).

## Consent

All participants read and signed an informed consent form agreeing to participate prior to the FGD.

## Conflicts of Interest

The authors declare no conflicts of interest.

## Supporting information

Annex_1.

## Data Availability

As per participants' signed informed consent form (see Annexe S2) and to maintain participants anonymity, individual participants data and focus group discussions audio recordings and transcripts will not be made available to others. In order to enable verifiability of the study results after completion of the project (12/2027), the data will be stored at the Technische Universität Berlin for a period of 10 years after project completion. Data access can only be granted in exceptional cases and upon reasonable request from the authors. Requests for data access should be addressed to the Data Protection Officer of Technische Universität Berlin via an email to the Department of Health Care Management of the Technische Universität Berlin: mig@tu-berlin.de. The received ethics approval from Newcastle University, the blank Informed Consent Form and the Interview guide used are available in the annexes.
