# Supplementary material for: Conceptualising Essential Oral Health Benefits Baskets: A Thematic Analysis of Public and Expert Perspectives
Source: Health Expect. 2025 Nov 20;28(6):e70501. doi: 10.1111/hex.70501 (PMC12634874; doi:10.1111/hex.70501)
Supplement: Supplementary file 1 — Annex_1. [file HEX-28-e70501-s001.pdf]

Annexes

Annex 1: Ethics Approval (Ref: 33388/2023) **2**

Annex 2: Informed Consent Form **3**

Annex 3: Open-ended Interview Guide for investigators chairing general population or experts focus group discussions (FGDs) **7**

Annex 1: Ethics Approval (Ref: 33388/2023)

Thank you for completing the University's Ethical Review Form. Based on your answers the University is satisfied that your project has met its ethical expectations and grants its ethical approval.

Please be aware that if you make any significant changes to your project then you should complete this form again as further review may be required. Confirmation of this decision will be emailed to you.

Please complete the declaration to submit your application.

Declaration

I certify that:

(11314)
Type: (M/multiple-opt)

[X]

the information contained within this application is accurate. (11441)

[X]

the research will be undertaken in line with all appropriate, University, legal and local standards and regulations. (11442)

[X]

I have attempted to identify the risks that may arise in conducting this research and acknowledge my obligation to (and rights of) any participants. (11443)

[X]

no work will begin until all appropriate permissions are in place. (11444)

Annex 2: Informed Consent Form

Informed Consent, Team Datenschutz der TU Berlin, Version vom 5.1.2023

Title of Research Project: PRUDENT - Prioritization, incentives and Resource use for sUstainable DENTistry

Principal Investigator: Dr. Ruth Waitzberg

Co Investigators: Béatrice Durvy, Lukas Schöner

Sponsor/Funding: the European Union, HORIZON2020

Project Duration: Jan. 2023 – Dec. 2028

1.Project Description:

Background & Purpose of Research: PRUDENT is a research study conducted by the Department of Health Care Management, TU Berlin in collaboration with international partners across the EU. PRUDENT aims to develop and implement an innovative and context-adaptive framework for public coverage and optimized financing of oral care. It brings together top investigators from prestigious universities, public authorities and policymakers, civil society and patient organizations, health insurers, and health professionals, to achieve a step change in collective problem solving. As part of the study, we are conducting focus group discussions with key informants.

Invitation to participate: You are being invited to participate in a focus group discussion as part of PRUDENT. This focus group discussion aims to learn the perspectives of different stakeholders such as the general population and oral health professionals on "essential oral health care". The ultimate goal is to build consensus on public preferences on oral health care coverage.

Procedures: If you decide to enrol in this study, you will participate in a focus group discussion, semi-structed in person or virtual (i.e., Microsoft Teams or Zoom) conducted by members of the research team. The focus group discussions will last approximately 1.5 hours.

Voluntary Participation & Early Withdrawal: Your participation in this study is voluntary. You can choose not to participate or refuse to answer a question, or withdraw at any time without penalty or loss of benefits to you. **If you wish to withdrawal from the study, you're data may still be used, unless you tell us otherwise.**

Risks: We do not foresee any risks.

Benefits:

You will be supporting an impactful EU project that aims to improve the coverage of oral health across European countries. Your participation helps to develop potentially broader coverage of and improved access to oral health services in the future. Participating in a large-scale EU project offers a unique opportunity to be part of macro-level decision-making, develop skills and gain exposure to EU initiatives. You can actively contribute your insights, experiences, and concerns related to oral health coverage and, with this, have a direct impact on policy decisions that affect your own oral health and that of your communities.

2. Information on the Planned Data Processing / Rights of Participants

The data generated as part of the project will be processed as follows:

- Conversations will be recorded using a dictation device.
- The audio recordings will be pseudonymized and stored on the secured servers of the Technische Universität Berlin.
- The audio recordings will be securely and irreversibly deleted from the dictation device.
- Access to the audio recordings will be restricted to project staff.
- The interview audio recordings will be transcribed in an anonymized form, and the transcripts will be pseudonymized. This means that all personally identifiable information will be anonymized to prevent any identification of individuals.
- The consent forms, contact information, translation key (pseudonymization), and the original audio recordings will be securely stored and kept separate from each other, ensuring that third parties cannot associate individuals with the audio recordings.
- The translation key will be deleted once the purpose of the research project allows it, which will be by December 31, 2028, thereby fully anonymizing the data. The consent forms with contact information will also be deleted at that time.
- The audio recordings and transcripts will be retained on the secured servers of the Technische Universität Berlin for ten years, in accordance with the guidelines for good scientific research, and then securely and irreversibly deleted.
- No data will be shared with third parties.
- The analysis and evaluation of the interviews will be based on the anonymized transcripts. The results may be used for scientific publications, including presentations at conferences and other scholarly publications. Only anonymized research findings that do not allow for identification of individuals will be published. Individual anonymized quotations may be included in publications.
- The anonymized transcripts may be archived and made available to other researchers.

- All project staff with access to personally identifiable data are bound by confidentiality and obligated to maintain data secrecy.
- Your consent to participate in the interview is voluntary. You will not face any disadvantages for choosing not to participate, and you may also refuse to answer specific questions.

According to the General Data Protection Regulation (DSGVO), you have the following rights:

- Confirmation of whether personal data concerning you is being processed (Article 15 DSGVO).
- Access to this data and information about the processing (Article 15 DSGVO).
- Correction of any inaccurate data (Article 16 DSGVO).
- Deletion of data if processing is no longer justified or required (Article 17 DSGVO).
- Restriction of processing in certain legally defined cases (Article 18 DSGVO).
- Transmission of your personal data, if provided by you, to yourself or a third party in a structured, commonly used, and machine-readable format (Article 20 DSGVO).
- Termination of processing and deletion of data that have already been generated after revocation of your consent (Article 21 DSGVO).

Please note that revocation can only occur until complete anonymization, as we will no longer be able to associate your data or delete it afterwards.

This project is in compliance with the data protection regulations of the XXXX. If you have any concerns regarding data protection in the project, please contact the project coordinators directly or:

Data Protection Officer of Technische Universität Berlin

Annette Hiller, K3-DS,

Straße des 17. Juni 135, 10623 Berlin,

E-Mail: info@datenschutz.tu-berlin.de

If you have any reason for complaint, you can also contact the responsible supervisory authority:

Berlin Commissioner for Data Protection and Freedom of Information

Alt–Moabit 59–61,10555 Berlin

E-Mail: mailbox@datenschutz-berlin.de

Publication of Research Findings: We will be preparing manuscripts for publication.

Compensation: In no way does signing this consent form waive your legal rights nor does it relieve the investigators, sponsors or involved institutions from their legal and professional responsibilities.

Participants will receive reasonable compensation for expenses and time.

3. Consent to Data Processing

Within the scope of the above-described project, we would like to process your personal data. Participation in the project is voluntary and is solely for scientific purposes. Therefore, we require your consent under Art. 6 Absatz 1 Nr. 1 der Europäischen Datenschutz-Grundverordnung (DSGVO).

Consent:

I agree to participate in the above-mentioned study. I have been informed about the study and have had the opportunity to ask questions. I understand that my participation in the study is voluntary, and I am free to withdraw without providing any reason, without suffering any disadvantages. The lawfulness of data processing prior to withdrawal remains unaffected.

I consent to our conversation being recorded on audio and transcribed verbatim. My statements will only be used for research purposes in anonymized textual form that does not allow for identification of my person. These anonymized transcripts may be archived and made available to other researchers within the XXXX consortium.

My personal data, particularly my name, phone number, and email address, will not be disclosed to third parties.

I have been informed about the project, as well as the procedures for evaluation, storage, and deletion of data (as described above) in writing.

Name and signature

, X

Location/Date Signature

Annex 3: Open-ended Interview Guide for investigators chairing general population or experts focus group discussions (FGDs)

1. Introduction

PRUDENT project:
As mentioned in the Informed Consent Form, by agreeing to participate in this session, you are agreeing to be recorded. If we are on Zoom, your video will be recorded as well. If you do not wish for your face to appear on the video, feel free to turn off your camera. If there is no objection, I will now start the record.

PRUDENT *(Prioritization, incentives and Resource use for sUstainable DENTistry)* is a 5-year EU-funded project which brings together investigators from The Netherlands, Germany, Portugal, Hungary, France, Estonia, Denmark, Ireland, Malta, Norway and the UK. The project aims to develop and implement a framework for countries wishing to improve coverage and financing of **oral health care** (OHC).
While oral diseases and conditions are the 3rd most expensive diseases to treat in the EU, many EU citizens have no access to oral health care without financial hardship.
Hence, in order to move the financing of oral health systems forward, PRUDENT uses a participative approach that is entirely focused on root causes underlying the current limitations of oral care financing.

You are being invited to take part in a focus group discussion as part of PRUDENT. This discussion aims to learn the perspectives of different stakeholders such as the general population and (oral) health professionals on “essential oral health care”. The ultimate goal is to build consensus on public preferences on oral health care coverage.

Starting after the introduction, the focus group discussions will last approximately 1H15.

You are divided into groups of 7 to 8 participants and presented with four different questions, as well as the opportunity at the end of the session to share any additional comment you may have. You are welcome to speak freely throughout the session. There is no right or wrong answer.
Please, follow the instructions given by the investigator who is chairing the discussion. No specific material is required for this FDG except the “Interview Material for FGDs with the General Population” provided by the investigator (on paper or via shared screen). Questions follow a specific order and should not be consulted ahead, so, if in-person, please refrain from going to the next page until you are invited to do so.

Additional information:

- This Informed Consent Form notably indicates that all the information collected during this study will be treated with strict confidentiality. Your identity will be kept confidential, and your personal information will not be linked to any responses or reported in any way that could identify you. The data collected will be pseudonymized and used for research purposes only and stored securely.

- The signed forms will be collected by the investigator at the start of the focus group discussion.

The Data Management Plan is available upon request to the investigators.

2. Questions

- *Welcome participants:*
    - *Present yourself and ask for participants to present themselves as well; please ask them to state their country of origin and age. No need to give their surnames.*
    - *Remind them that there is no right or wrong answer, that we want to hear everyone, inform them that specific durations were allocated per question, and ask them to be mindful of the microphone (avoid any side noise).*
- *As a general rule, the interview chair should provide as little additional information as possible. Yet, for each question, some probe questions are included in the interview guide, to use if participants find it difficult to give an answer or do not respond at all. If needed, please ask the questions following the proposed order.*

1. From your perspective, what is essential when it comes to your general health?

Duration: 15 minutes

- *Open question.*
- *Let participants answer on a voluntary basis at first, but then make sure every participant was given the opportunity to contribute.*
- *Be mindful of the time.*

If needed, please refer to the following probe questions:

- ***Question 1:** Can you think of certain attributes that have to be fulfilled to make a health service essential?*
- ***Question 2:** If you think of a specific personal experience where you felt that a certain health service was essential, what made it essential in that situation?*

2. How would you define “essential oral care”?

Duration: 15 minutes

- *Open question.*
- *Let participants answer on a voluntary basis at first, but then make sure every participant was given the opportunity to contribute.*
- *Be mindful of the time.*

If needed, please refer to the following probe questions:

- ***Question 1:** Imagine you are deciding on an essential list of oral health service to be reimbursed, based on which attributes would you define “essential”?*
- ***Question 2:** If you think about* general health as in *the first question, to what extent does it apply to oral health services?*
- ***Question 3**: If you list individual services, try to think about what exactly made these services essential.*

Duration: 2 minutes to present the table and let them go quickly through it.

There are various ways of grouping or categorising Oral Health (OH) services. For the purpose of this research, we are using the groupings depicted in Table 1.

Table 1. Types of OH benefits and their associated OH services and procedures.

Type of benefit	Services and procedures
Population-wide and self-care prevention measures	Community water fluoridation, salt fluoridation, fluoridated toothpaste, and maintaining oral hygiene
Emergency and urgent oral health care	Infection, swelling, pain, or serious bleeding
Diagnostic and preventive oral services	Early detection, X-rays (bitewing, periapical, full-mouth), Oral cancer screening, Removal of plaque, calculus and stains from the tooth structures, Fluoride application (varnish, gel)<br>Fissure sealant, Oral hygiene, Dietary or smoking cessation advice
Treatments for the most prevalent oral health problems	Most curative and basic restorative services including fillings and root canals, extractions, oral and maxillofacial surgery
Advanced oral health care	• Prosthetic or orthodontic services<br>• Major restorative care, including dentures, bridges, inlays/onlays, and crowns
Cosmetic dental services	Teeth whitening, tooth bonding, Dental veneers

Source: Winkelmann et al (2022) Oral health care in Europe –Health in Transition (HiT) Review 2022 *https://tinyurl.com/OBSoralhealthHIT*

Based on the table above:

3. **Among the following groups of services, select the ones you consider essential. Explain why you consider them essential.**

Duration: 14 minutes.

- *Participants are allowed to take notes if they want to.*
- *Open question.*
- *Let participants answer on a voluntary basis at first, but then make sure every participant was given the opportunity to contribute.*
- *Be mindful of the time.*

If needed, please refer to the following probe questions:

- ***Question 1:*** *Which of these services do you consider essential and why?*
- ***Question 2:*** *Which criteria come to your mind while choosing these services?*
- ***Question 3:*** *And if you had to prioritise some services, which criteria come to your mind to choose among these services? (to use in case they are selecting all the services)*

4. **Who would you cover for these services under limited resources? Why?**

Duration: 14 minutes.

- *Participants are allowed to take notes if they want to.*
- *Open question.*
- *Let participants answer on a voluntary basis at first, but then make sure every participant was given the opportunity to contribute.*
- *Be mindful of the time.*

If needed, please refer to the following probe questions:

- *Question 1: Assuming you are advising the health policy makers from your country, which services would you prioritise for which population groups?*
- *Question 2: Which types of services are essential for whom? How do we define that?*

5. **Is there anything you would like to add? Or is there any question we didn't ask and we should have asked?**

Duration: 10 minutes.

3. Conclusion

Duration: 5 minutes.

Many thanks for your participation in this study. During this exercise, we gathered information to:

- To conceptualise what is an 'essential' OH service; and
- To better understand your perspective and preferences regarding the **scope** (meaning the type of services reimbursed) and the **breadth** (meaning the population who can benefit from reimbursement) of OH services reimbursement.

This data will be included in the results from other FGDs performed among other groups with (oral) health professionals and the general population, within other PRUDENT partners' countries.

Take away message:

While in many countries, there is no comprehensive coverage for OH services and many patients around the world still experience financial hardship related to OHC, good oral health entails much more than only good teeth as described in the WHO definition of Oral Health (Box 1).

Box 1. The WHO definition of Oral Health.

"Oral health is the **state of the mouth, teeth and orofacial structures** that enables individuals to **perform essential functions** such as eating, breathing and speaking, and **encompasses psychosocial dimensions** such as self-confidence, well-being and the ability to socialize and work without pain, discomfort and embarrassment. Oral health varies over the life course from early life to old age, is **integral to general health** and supports individuals in participating in society and achieving their potential."

Source: World Health Organization. Oral health – overview. Available at: https://www.who.int/health-topics/oral-health#tab=tab_1
